# Catecholaminergic modulation of trust decisions

**DOI:** 10.1007/s00213-019-5165-z

**Published:** 2019-01-31

**Authors:** Cătălina E. Rățală, Sean J. Fallon, Marieke. E. van der Schaaf, Niels ter Huurne, Roshan Cools, Alan G. Sanfey

**Affiliations:** 1 Rotterdam School of Management, Erasmus University Rotterdam, Rotterdam, 3062 PA The Netherlands; 2 Donders Institute for Brain, Cognition and Behaviour, Centre for Cognitive Neuroimaging, Radboud University, 6500 HB Nijmegen, The Netherlands; 3 Department of Psychiatry, Radboud University Medical Centre, Nijmegen, The Netherlands; 4 Karakter Child and Adolescent Psychiatry University Centre, Nijmegen, The Netherlands; 5 Behavioral Science Institute, Radboud University, Nijmegen, The Netherlands

**Keywords:** Methylphenidate, Ritalin, Trust Game, Trust, Social learning, Decision-making, Catecholamines

## Abstract

**Rationale:**

Trust is a key component of social interactions. In order to assess the trustworthiness of others, people rely on both information learned from previous encounters, as well as on implicit biases associated with specific facial features.

**Objective:**

Here, we investigated the role of catecholamine (dopamine and noradrenaline) transmission on trust decisions as a function of both experienced behavior and facial features.

**Methods:**

To increase catecholamine levels, methylphenidate (MPH, i.e., Ritalin®, 20 mg) was administered to participants (*N* = 24) prior to their playing a well-studied economic task, namely the Trust Game (Berg et al. 1995). We measured the amount of money invested with a variety of game partners. Across game partners, we manipulated two aspects of trust: the facial trust level (high facial trust, low facial trust, and non-social) and the likelihood of reciprocation (high, low).

**Results:**

Results demonstrated no main effect of MPH on investments, but rather a selective lowering of investments under MPH as compared with placebo with the game partners who were low on facial trustworthiness and were low reciprocators.

**Conclusion:**

These results provide evidence that MPH administration impacts social trust decision-making, but does so in a context-specific manner.

## Introduction

We engage in decision-making on a daily basis: from relatively unimportant choices, such as deciding what to wear on a particular day, to ones of greater consequence, such as whether to accept a particular job offer. Though some of our decisions are made outside of any specific social context (e.g., what songs to put on a workout playlist), many of our choices consider, to a greater or lesser extent, the feelings, beliefs, expectations, and behaviors of our social partners (e.g., choosing a Christmas present for a friend).

These latter social choices are well exemplified by the decision to trust another person. Trust is often defined as the willingness to rely on another individual, with the associated risk of that trust being abused. To this end, deciding on whether a social partner is worthy of trust involves an estimation of the likelihood of being betrayed. This important process is typically studied experimentally using the Trust Game (Berg et al. 1995), a well-characterized financial investment task that allows the assessment of the various factors that can influence trust decisions. Investment amounts in the Trust Game have been shown to vary with several factors, such as knowledge or beliefs about the moral character of the game partner (Delgado et al. 2005a), perceived trustworthiness of the game partner (Van ‘t Wout and Sanfey 2008), experienced behavior (King-Casas et al. 2005, Krueger et al. 2007), and expectations about general investment patterns in the Trust Game (Chang and Sanfey 2013).

In essence, trust decisions are concerned with the assessment of beliefs about a partner’s future behavior. Previous literature has shown that trust decisions are often reliant on information about the past behavior of our interaction partner, such as reputation (Axelrod and Hamilton 1981; Delgado et al. 2005a; King-Casas et al. 2005). In addition to using past behavior to inform future behavior, inferences are made about a person’s character based on invariant facial features, such as the size of the eyes or the width of the cheekbones (Winston et al. 2002; Willis and Todorov 2006). Research has shown how both the facial features and the past behavior of a social partner interact in order to construct trust beliefs (Chang et al. 2010). This was demonstrated by employing a reinforcement learning framework where social learning of trust depended on rewards (i.e., the positive experience of trust reciprocation) and punishments (i.e., the negative experience of trust abuse). Thus, two key components of trust are the ability to infer an initial impression of the reliability of others, as well as the ability to learn from subsequent positive and negative experiences.

Learning from rewards and punishments is well established to depend on catecholamine transmission, and more specifically on striatal dopamine (DA) (Collins and Frank 2013). Striatal DA has also been shown to modulate probabilistic reinforcement learning (Bódi et al. 2009; Frank et al. 2004; Pessiglione et al. 2006). Moreover, work from Doll et al. (2011) has shown that DA can influence the degree to which prior instructions and actual experience interact and bias choice. However, whether these effects generalize into affecting aspects of higher order social cognition has yet to be investigated. Here, we examine the effects of a catecholamine challenge on trust-based decisions by assessing effects of acute administration of a single oral dose of methylphenidate (MPH) on performance in a repeated Trust Game.

MPH is a psychostimulant drug that efficiently elevates the extracellular levels of DA in the striatum (Volkow et al. 2005) as well as DA and noradrenaline (NA) in the prefrontal cortex. It acts by blocking the DA and the NA transporter and thus elevates catecholamine levels in the synapse. MPH has been shown to increase affective and neural responses to reward in healthy adults (Volkow et al. 2002), to potentiate learning from reward versus punishment (Clatworthy et al. 2009; Tye et al. 2010; Frank et al. 2011; van der Schaaf et al. 2013) and to boost cognitive performance (Arnsten 2011; Swanson et al. 2011). Thus, one potential route for MPH to impact trust could be by modulating learning directly from rewards and punishments respectively.

However, as noted above, trust involves more than just learning from rewards and punishments. As shown by Chang et al. (2010), a defining feature of trust is the use of both current feedback as well as prior beliefs to inform future behavior. Instructions or inferred moral character have a strong effect on subsequent behavior and learning (Delgado et al. 2005a, b; Doll et al. 2009). Therefore, MPH might also impact trust decisions through altering initial beliefs about the partner’s inherent moral character, which is often inferred directly from facial features. Doll et al. (2011) have shown that the degree to which initial biases are strengthened by experience is linked to genetic dopaminergic gene variations. Relatedly, ter Huurne et al. (2015) have demonstrated that MPH increases the attention to faces over scrambled images, making it plausible that these social factors may be enhanced under MPH. Moreover, MPH has previously been found to impact decision-making, particularly in a social context (Campbell-Meiklejohn et al. 2012a). Therefore, another potential route for MPH to impact trust could be via these social factors.

Thus, we predict that MPH will have an adaptive impact on trust decisions. Specifically, we hypothesize that, in terms of the likelihood of reciprocation of a given partner, there will be a decrease in investments with low reciprocating partners and an increase in investments with high reciprocators. Additionally, we hypothesize that social factors may also play a role, especially when interacting with a game partner for the first time, in which case MPH could enhance the salience of facial trustworthiness. Finally, as discussed above, a number of studies have demonstrated that these two factors, learning and facial trustworthiness, interact and therefore we will also explore how MPH may modulate this interaction.

In order to investigate the role of catecholamines on trust, we performed a double-blind, placebo-controlled, crossover design. Following drug administration, participants played a well-known economic task designed to quantify trust decisions, namely the Trust Game (Berg et al. 1995). Our version of this task enabled us to disentangle the influence of catecholamines on trust decisions that could be driven by two main factors. The first, an initial social bias, was examined by pairing participants with game partners who possessed differing levels of facial trustworthiness. The second, learning via direct interaction, was explored by playing with partners who demonstrated varying likelihoods of reciprocating the participant’s trust. Finally, and importantly, we investigated the interaction of these two factors, in order to capture any condition-specific effects.

## Materials and methods

### Participants

Twenty-four students (11 men, *M* age = 20.60; *SD* = 2.20) of the Radboud University participated in this experiment. The participants gave written informed consent and received monetary reward in return for taking part in the study. The study was approved by the local medical-ethical committee (Committee for Protection of Human Subjects of the Arnhem/Nijmegen region; CMO protocol number 2010/283) and adhered to the guidelines of the Declaration of Helsinki.

### Screening

All participants were healthy volunteers and had no relevant history of medical or psychiatric conditions and no history of drug abuse. Participants were not accepted in the study based on the following criteria: fulfillment of ADHD criteria, family history of schizophrenia, bipolar disorder, depression or neurological abnormalities. They had to be currently free of over-the-counter medication use, drink less than 20 units of alcohol, and smoke less than 20 cigarettes per week. If cannabis was used less than 2 weeks prior to testing or if there was a history of frequent use of recreational drugs (more than 5 times weekly), the volunteers were not allowed to participate in the study. During a first intake session, all participants were screened by both a medical doctor and by a psychologist. This procedure consisted of a physical examination of weight, heart rate and blood pressure, a medical examination, and the Mini-International Neuropsychiatric Interview (MINI) (Sheehan et al. 1998).

### Pharmacological manipulation

A within-subjects, double-blind, placebo-controlled crossover design was employed. Participants were administered MPH (Ritalin®, 20 mg) and placebo on two separate experimental sessions, at least 1 week apart from each other. All participants were asked to not consume alcohol or take any medication 24 h prior to testing and to not consume caffeine on the day of testing. The current task was part of a larger protocol: it was preceded by a functional magnetic resonance imaging (fMRI) experiment and two short behavioral experiments reported elsewhere (Fallon et al. 2017; van der Schaaf et al. 2013; ter Huurne et al. 2015). The task was executed ~ 3 h after drug intake and lasted for ~ 17 min. Although time of testing was optimized for the preceding fMRI paradigm (Fallon et al. 2017), assessment of the current task coincided with the active time window of drug effects (~ 4 h with peak plasma levels 1½ hour) (Volkow et al. 2002; Swanson and Volkow 2003).

### The Trust Game paradigm

**Fig. 1:**
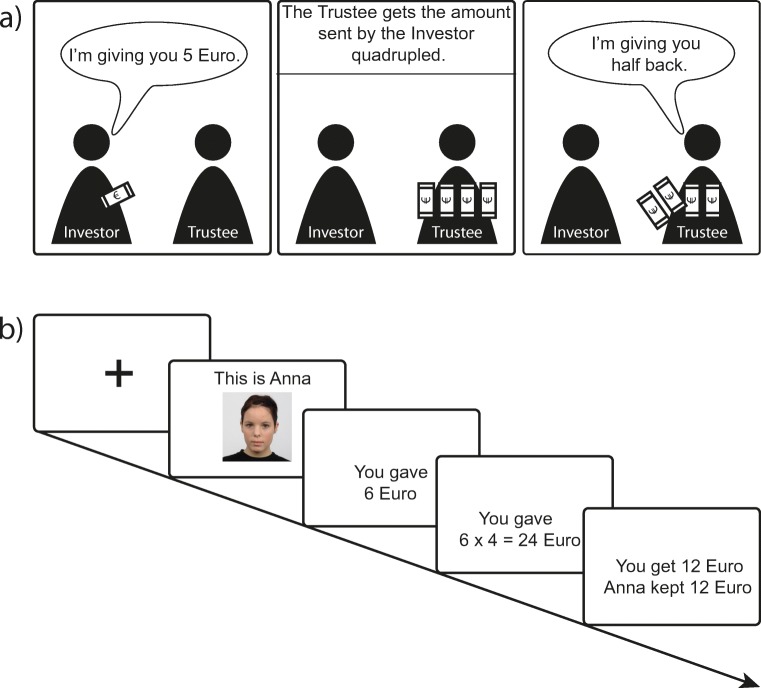
**a** The Trust Game. There are two players in the game, Investor and Trustee. The Investor is provided with a 10 Euro endowment. From this endowment, the Investor must choose how much money they will send to the Trustee, with this being any amount from 0 to 10 Euros, in increments of 1 Euro. The money transferred is multiplied by a factor of 4 by the experimenter, such that the Trustee receives this quadrupled amount. Finally, the Trustee decides if they want to send any money back to the Investor, with no requirement that any money is returned. **b** This represents the time course of a single trial within the experiment. Each square represents a screen. A fixation cross was shortly presented, followed by the face and the name of the game partner for that trial (3000 ms). The participant could then decide how much they wanted to invest in the game partner, by pressing a button to increase the investment amount in 1 Euro increments. When the participant was satisfied with their choice, they pressed an additional button to finalize the investment. If no offer was submitted in time (6000 ms), all of the money for the round was forfeited. The final feedback screen informed the participant whether their game partner had shared or kept the money. The length of a trial varied between 13 and 16 s

Figure 1a and b show the experimental task design. In order to address our research question, we used a modified version of the Trust Game. Participants played a repeated Trust Game in the role of the Investor. There are two players in the game: Investor and Trustee. The Investor is provided with a 10 Euro endowment. From this endowment, they must choose how much money they will send to the Trustee (any amount from 0 to 10 Euros, in increments of 1 Euro). The Investor keeps the money not transferred. The money transferred is multiplied by a factor of 4 by the experimenter, such that the Trustee receives this quadrupled amount. Finally, the Trustee decides whether to send any money back to the Investor, with no requirement that any money is returned. Several studies show that, in a one-shot game, Investors send on average approximately half of their initial endowment (Camerer 2003).

Here, to investigate trust behavior across several interactions, participants played a repeated version of the Trust Game, meaning they had multiple exchanges with each of the game partners. On each trial, participants first saw a photo of their game partner on the screen, and then decided how much money, if any, they would transfer to the respective partner. Next, they received feedback about the decision of their partner, with the amount transferred presented on the screen.

Within a trial, a fixation cross was first presented, followed by the picture and the name of the partner for that round (3000 ms). The participant could then decide how much they wanted to transfer by pressing a button to increase the investment amount in 1 Euro increments. When the participant was satisfied with their choice, they pressed a separate button to confirm their investment. To ensure that the number of button presses was orthogonal to the investment amount, the starting investment value on the screen was randomly generated between 0 and 10. If the participant did not submit an offer within 6000 ms, they forfeited all of their money for the round. The final feedback screen informed the participant whether their game partner had shared or kept the money (which we manipulated across trials, see below), also displaying the respective amounts (4000 ms). The total length of a trial varied between 13 and 16 s.

#### Dependent variable

We used the amount invested with a game partner as the dependent variable. On each trial, a participant could invest any amount from 0 to 10 Euros, in increments of 1 Euro.

#### Independent variables

We assessed the effect of two factors on trust behavior: the effect of prior social biases and the effect of experience with a game partner. Importantly, we were particularly interested in the interaction of these two factors.

To investigate the effect of social biases on trust decisions, we manipulated the type of partner with whom the participant interacted. Participants played with two types of ostensible human partners: one which was rated as high in facial trustworthiness and one rated as low in facial trustworthiness (Oosterhof and Todorov 2008) (see details in *Game partners*). Additionally, for examining the effect of social versus non-social information on trustworthiness, participants also interacted with a non-social partner, namely a slot machine.

To capture the effect of experience, we manipulated the likelihood with which a partner would reciprocate trust—participants played with three high reciprocity partners (two human and one slot machine) and three low reciprocity partners (also two human and one slot machine). Therefore, participants interacted with a total of six game partners, namely: one with high facial trust and high reciprocity, one with high facial trust and low reciprocity, one with low facial trust and high reciprocity, one with low facial trust and low reciprocity, one slot machine with high reciprocity, and one slot machine with low reciprocity. If an offer was reciprocated, the game partner always reciprocated by giving back half of the total multiplied amount sent by the participant. When an offer was not reciprocated, the game partner did not return any money to the participant. On each trial, the game partner’s decision to reciprocate was drawn from a distribution centered around a probability of reciprocation of 75%. Similarly, in the low reciprocation the decision to reciprocate was drawn from a distribution centered around a probability of reciprocation of 25%.

Both partner type and probability of reciprocation were within-subject measures. Drug administration was a within-subject factor, such that each participant was given both placebo and MPH. The order in which these were administered was counter-balanced, resulting in a between-subject measure of order, either placebo-MPH (12) or MPH-placebo (12).

#### Runs and trials

As mentioned above, participants were tested on two separate occasions: on one they were administered MPH, on the other placebo. On each of these occasions, participants completed a session of 72 trials of the Trust Game, viewing another stimulus set of four faces each time. Each experimental session was divided in three runs, in which trials appeared evenly distributed in pseudo random order. Overall, they played 12 times with each game partner. Each run had a total of 24 trials, four trials with each of the six game partners (two high-trust faces, two low-trust faces, two slot machines).

#### Game partners

The stimuli were eight frontal, colored photographic images of emotionally neutral faces and two pictures of slot machines. Pictures showing faces were selected from a standard set of 60 images (the Radboud Faces Database) and were controlled for low-level visual features. The faces were selected on the basis of trustworthiness ratings on a 7-point Likert scale, which were given by 98 healthy subjects in a pilot study. Since each participant underwent two experimental sessions, we used two different sets of pictures. Each set consisted of four faces (two men, two women) and two slot machines. The trustworthiness levels within each set were matched such that one man and one woman had high trustworthiness (*M* = 5.20, *SD* = 0.76) and the other man and woman had low trustworthiness ratings (*M* = 3.10, *SD* = 0.51). E-Prime software (Psychology Software Tools, Inc., Pittsburgh, PA) was used for stimulus presentation.

### Statistical analysis

All behavioral data analyses were performed using the R statistical package, version 3.5.1 (R Development Core Team 2018) within RStudio, version 1.1.442.

We wanted to address several research questions. Firstly, we were interested in testing whether MPH administration impacts trust behavior overall, as measured by the mean amount invested in the game partner. Secondly, we designed our task specifically to explore whether MPH could impact the initial beliefs about the facial trust level of a partner. Additionally, we wanted to compare whether there is a social versus non-social effect of MPH. Thirdly, we included several trials per game partner, together with two levels of reciprocation. This allowed us to investigate whether the likelihood of reciprocation has different effects on the amounts invested, as a function of drug administration. Finally, we wanted to address whether the facial trust factor interacts with the reciprocation factor, and if this effect responds differently to the catecholamine challenge.

To address these questions, we ran a trial-by-trial mixed effects model. The model was estimated using *lme4* (Bates et al. 2015), called using the lmer function of the package *lmerTest*, version 2.0-36, in R. Our model had the following fixed factors: drug administration (placebo, MPH), facial trust level (high facial trust, low facial trust, slot machine), and reciprocation level (high, low), together with all their interactions. We had random intercepts per participant, and random slopes for drug administration, game partner, and probability of reciprocation (Baayen et al. 2008). Using mixed effects models allowed us to best account for non-independence in the data, without aggregating data points (i.e., investment decisions) and thus reducing statistical power. Moreover, using both random slopes and random intercepts within the mixed effects models enabled us to consider individual variation in the investment choices as a function of drug administration, partner likelihood of reciprocation, and partner facial trustworthiness.

Furthermore, we tested whether there were any order effects in our experiment, by adding the between subjects fixed factor “order” to the mixed model, to check for test-retest effect of the iterated Trust Game. The single-shot Trust Game has no test-retest effects. However, this repeated version is designed specifically to trigger learning about the trust behavior of the game partner. Optimal learning in this task entails tracking of reciprocation probabilities and adjusting the investment behavior accordingly.

For specifically investigating the effect of the initial beliefs about trustworthiness and how MPH impacts that process, we ran an ANOVA, using the ezANOVA function of the *ez* package, version 4.4-0. We used only the first game interaction with each of the game partners (i.e., the first trial in each condition). This served a double function: first, it ensured that each partner type was treaded as intended (i.e., the faces that were used as proxies for higher trustworthiness actually received higher initial investments), and second, it allowed us to investigate whether drug administration altered the perception of these facial features.

*p* values were computed using type II chi-square tests as implemented in the ANOVA function. Post-hoc comparisons were performed using the testInteractions function of the *phia* package, version 0.2-1, and were Holm-corrected for multiple comparisons.

## Results

### First trial

**Fig. 2:**
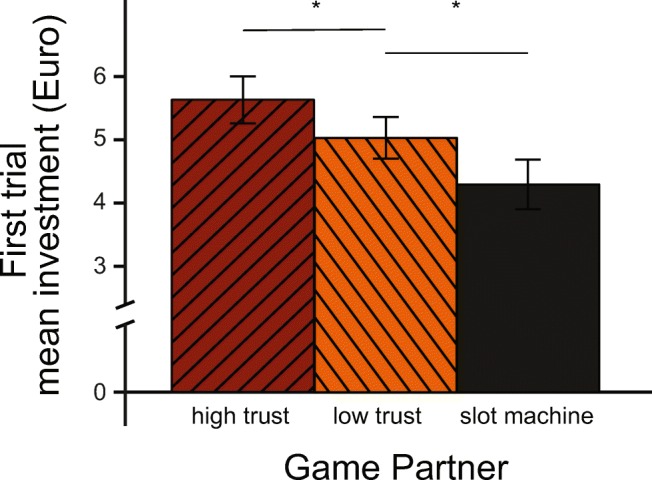
The figure illustrates the mean investments (± SE) with the three levels of facial Trust Game partners, in the first trial of the Trust Game only

One set of analyses focused only on the first encounter (trial) between participants and each of the game partners, to assess whether facial trustworthiness alone had an impact on investment amounts, and whether methylphenidate modulated this effect. We found a main effect of facial trustworthiness expressed in an overall significant difference in mean investments on those trials (Facial Trust, *F*(2, 271) = 5.896, *p* = 0.003). Pairwise comparisons revealed significant differences between all three types of facial trustworthiness: participants invested more with high facial trust partners as compared to low facial trust partners (*M* diff = 0.684; *SE* = 0.287; *p* = 0.026), and less with slot machine partners, as compared to both high and low facial trust partners (*M*_SlotVSHighTrust_ diff = − 1.615; *SE* = 0.407; *p* = 0.001; *M*_SlotVSLowTrust_ diff = − 0.961; *SE* = 0.353; *p* = 0.015, see Fig. 2). We found no main effect of drug (Drug, *F*(1, 271) = 0.174, *p* = 0.677), and no interaction with drug (Drug × Facial Trust, *F*(2, 271) = 0.381, *p* = 0.684). Thus, while facial trustworthiness had an effect on the initial investments, MPH did not alter those patterns.

### All trials

The second set of analyses used all the task trials in a mixed-model approach. We examined whether MPH could impact the initial beliefs about the facial trust level of a partner, the effect of experience with a partner, and their interaction.

Across all trials, facial trust level impacted investments (Facial Trust, *χ*^2^(2) = 10.286, *p* = 0.006). However, this main effect was driven by the difference between human faces and slot machines: high facial trustworthiness versus slot machine (*M* diff = 0.814; *SE* = 0.272; *p* = 0.008) and low facial trustworthiness versus slot machine (*M* diff = 0.718; *SE* = 0.289; *p* = 0.035). There were no significant differences between the two levels of human facial trustworthiness (*M* diff = 0.095; *SE* = 0.191; *p* = 0.871).

There was a trend for the impact of drug administration on investments as a function of facial trustworthiness across the entire experiment (Drug × Facial Trust, *χ*^2^(2) = 5.160, *p =* 0.076).

Over the course of the experiment, participants learned about the likelihood of game partners to reciprocate their investment. This was reflected in a main effect of reciprocity on amount invested, where participants invested more with game partners who were high reciprocators than with low-reciprocating partners (Reciprocation, *χ*^2^(1) = 111.431, *p* < 0.001). However, knowledge about the likelihood of reciprocation was not affected by drug administration (Drug × Reciprocation, *χ*^2^(1) = 0.003, *p* = 0.956).

**Fig. 3:**
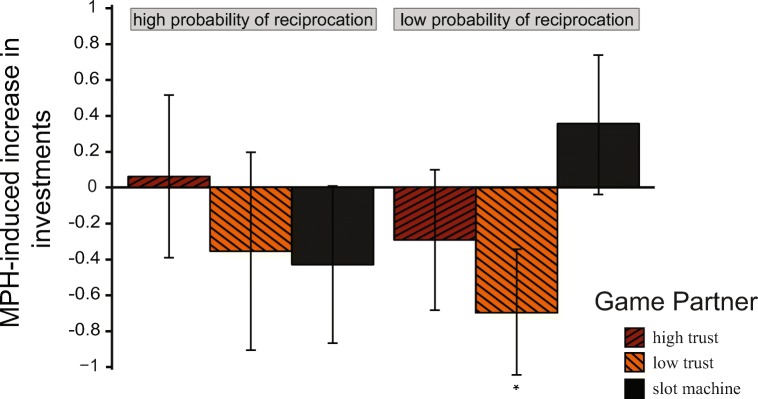
The figure presents the three-way interaction of Drug × Reciprocation Probability × Facial Trust. The *y*-axis shows the difference in investment amounts between MPH and placebo (± SE)

Next, we explored the drug effects on the interaction between facial trustworthiness and reciprocation. This key three-way interaction was significant (Drug × Facial Trust × Reciprocation, *χ*^2^(2) = 8.109, *p* = 0.017), and it was driven by the different drug effects for slot machine partners, on the one hand, and the human partners, on the other, when comparing high versus low reciprocation likelihood (High Trust vs Slot Machine, *χ*^2^(1) = 6.287, *p* = 0.037, and Low Trust vs Slot Machine, *χ*^2^(1) = 5.892, *p* = 0.037 respectively, see Fig. 3). There was no difference between the high and low trust game partners (*χ*^2^(1) = 0.007, *p* = 0.936). Specifically, we found a significant simple main effect: for low reciprocators, investments with the low trust partners were lower on drug as compared with placebo (*χ*^2^(1) = 7.476, *p* = 0.038), while this effect was missing for the slot machine and for the high trust partners (Slot Machine, *χ*^2^(1) = 1.673, *p* = 0.677, and High Trust, *χ*^2^(1) = 1.335, *p* = 0.677). In contrast, for high reciprocators, investments with high trust, low trust, and slot machines showed no effects of drug (High Trust, *χ*^2^(1) = 0.117, *p* = 0.733; Low Trust, *χ*^2^(1) = 1.889, *p* = 0.677; Slot Machine, *χ*^2^(1) = 2.869, *p* = 0.452). Together, this indicates that MPH decreased investment amounts specifically with low trust social partners that did not reciprocate often.

The interaction between facial trustworthiness and reciprocation likelihood was significant both under placebo (Placebo: Facial Trust × Reciprocation, *χ*^2^(1) = 415.74, *p* < 0.001), and under MPH (MPH: Facial Trust × Reciprocation, *χ*^2^(1) = 517.79, *p* < 0.001).

Finally, we found that participants invested on average about 50% of their allocated amount, and investments with ostensible human game partners were on average higher than those with slot machines (*M* diff = 0.800; *SE* = 0.263; *p* = 0.004). Mean investments per each experimental condition are reported in Table 1.

**Table 1:** Mean invested amounts across drug, reciprocation probability, and facial trust (means and standard deviations)

Mean investment amounts with each game partner (euro)		Drug administration	
		Placebo	MPH
High reciprocation	High Trust	6.50 (2.18)	6.74 (1.74)
	Low Trust	6.51 (1.69)	6.16 (2.07)
	Slot Machine	5.03 (2.66)	4.73 (1.98)
Low reciprocation	High Trust	3.00 (1.76)	2.69 (1.47)
	Low Trust	3.29 (1.55)	2.58 (1.12)
	Slot Machine	2.79 (1.63)	3.12 (1.69)

### Order effects

Given that participants played the Trust Game twice across two different sessions, we examined whether order effects could account for the drug effects outlined above. When controlling for drug administration order, the significance and interpretation of reported effects were not altered, although we found a main effect of the order of drug administration (Order, *χ*^2^(1) = 4.726, *p* = 0.030). To further ensure the reliability of the task results, we compared only the first session across groups, thus eliminating the order factor. Here, we again observe a reliable effect of Drug × Facial Trust × Reciprocation (*χ*^2^(2) = 11.885, *p* = 0.003). Moreover, the simple main effect of a decrease in investments with low reciprocators who were low on trustworthiness was significant (*χ*^2^(1) = 5.7019, *p* = 0.017).

## Discussion

The current study illustrates how investment behavior in a well-characterized economic exchange task, the Trust Game, can be altered by a catecholamine challenge.

Our results are consistent with several previous findings regarding investment behavior in the Trust Game. Participants invested on average slightly less than half of their endowment with their game partners, in line with previous literature (Fehr and Schmidt 1999; Camerer 2003; Camerer and Fehr 2006). As expected, in the first interaction with each game partner, the partner’s facial trustworthiness influenced investments, specifically, game partners with high facial trustworthiness received more money on this initial interaction than did those with low facial trustworthiness (Van ‘t Wout and Sanfey 2008; Chang et al. 2010). However, the influence of facial features on investment amount was not significant across all game interactions with a given partner, that is, the influence of the partner’s facial features diminishes over time on average. Additionally, the behavior of the game partners, in terms of how likely they are to reciprocate the participant’s trust, influences investments: high-reciprocating game partners receive larger investments than low-reciprocating partners (Singer et al. 2004; King-Casas et al. 2005), demonstrating that participants can learn these probabilities across the task. Furthermore, investments in slot machine partners were lower than investments with the human game partners.

The main focus of this study was on how administration of MPH would impact these effects. Contrary to our hypothesis, when compared with placebo, MPH did not alter investment patterns on the first encounter with a partner. There was also no effect of drug on investment amount as a function of the likelihood that a particular partner would reciprocate. However, drug administration did interact with facial trustworthiness and with the probability of reciprocation. We found a selective effect of drug on investment behavior towards those partners with both low facial trustworthiness and a low probability of reciprocation. Specifically, players invested less in low-reciprocating, low trustworthy-looking game partners when given MPH compared with placebo.

This study is among the few to investigate how catecholamines may alter decision-making in social contexts, and is novel in focusing on iterated trust behavior. Our finding that MPH affects investment when playing with a particular set of game partners, namely those who were low in trust and low in reciprocation, is supported by previous work examining the role of MPH in social processing. For example, Campbell-Meiklejohn and colleagues (2012a), demonstrated that administration of MPH influenced behavior in a social rating task, finding an increased salience of the social judgments of others. Here, the authors theorized that MPH is likely to increase interest and motivation during cognitive tasks, via the mechanism of increasing extracellular dopamine levels in the striatum. The idea that MPH can increase selective motivation is further supported by several studies showing how acute MPH (and amphetamine) administration can facilitate the identification of facial expression of emotions in healthy subjects (Wardle and De Wit 2012; Hysek et al. 2014). In a similar vein, though only examined in children with ADHD, MPH also enhanced empathy and theory of mind abilities (Maoz et al. 2014). More evidence of specific effects of elevated DA in a social context comes from Eisenegger et al. (2014), who showed that L-DOPA administration affects participants’ investment choices as a function of endogenous dopamine levels, as assessed by the DAT1 genotype. Finally, in a related study, Pedroni et al. (2014), showed that a single dose of L-DOPA can lower investments in a social economic game, though only when participants are not under the threat of punishment. These latter findings are explained as a consequence of a shift in focus: participants become more focused on personal monetary gains under L-DOPA, when the social norms cannot be enforced.

Notably in our study, MPH had an effect on partners who were lowest in terms of both facial trustworthiness and reciprocation. Interestingly, a previous study found that a high dose of MPH (60 mg) increased the recognition of sad and fearful faces particularly (Hysek et al. 2014), and also increased anxiety levels compared with placebo. Other research has found that MPH led participants to enhance the recognition of anger and fear in subjects with ADHD (Williams et al. 2008), and also resulted in participants more often misclassifying an emotion as anger (Dolder et al. 2018). Relatedly, a similar negative bias in the processing of emotionally laden stimuli was reported under higher doses of amphetamine (Wardle and De Wit 2012). As both MPH and amphetamine stimulate the DA and NE systems, our findings suggest that elevated MPH may be linked to a negative bias in processing of socially relevant stimuli, but specifically in the context of negative experience.

One mechanism that could underlie this observed drug effect is that of an increase in confirmation bias (Doll et al. 2011). Confirmation bias refers to the persistence of behaviors that are in line with the described, but not with the experienced, contingencies (Nickerson 1998). That is, experiences which are congruent with prior beliefs receive higher weight than experiences which are incongruent with prior beliefs or expectations. For instance, during election season, people are more likely to believe positive information about their favorite candidates, while at the time disregarding negative information about them. Thus, this account, as per our hypothesis, would predict that under MPH, partners with low facial trustworthiness and low reciprocation would evoke a decrease in investments, while those with high facial trustworthiness and high reciprocation would elicit an increase in investments, both relative to placebo. Our results do exhibit the former pattern, though not the latter. One possible explanation for the lack of confirmation bias in the positive direction is that there may be a ceiling effect in investments with this latter group, namely, that investments in this game seldom reach the maximum level, especially as “high reciprocators” only reciprocate in approximately 75% of the interactions. We in fact did find a difference in investment amounts, with more being sent to the high facial trustworthiness, high reciprocation group under MPH than placebo, in accordance with a confirmation bias account, though this difference did not reach significance. The selective reduction in investments only for the low facial trust low reciprocation game partners suggests an increase in the salience of prior beliefs on the subsequent value-based learning.

An alternative explanation for our results could be a change in risk perception as a function of drug administration. In clinical populations, MPH has been linked to riskier decision tendencies compared with healthy individuals (Rahman et al. 2006; DeVito et al. 2008; Shiels et al. 2009). In healthy participants, Campbell-Meiklejohn et al. (b) showed that MPH altered risk-taking in a gambling task as a function of the size of the stake. In particular, with small stakes (similar in magnitude to those used in the current study), under MPH participants demonstrated a trend towards increased risk aversion, that is, a tendency not to gamble. This is consistent with the behavior of the low facial trust low-reciprocating game partners in our study. However, in their study, MPH also led to an increase in risky behavior with larger stakes, suggesting a more complex relationship between MPH and risk attitudes. Given that MPH has quite a high frequency of use not only in clinical population (ADHD), but also in healthy individuals (Smith and Farah 2011), it is important to investigate how this drug impacts higher cognitive functions, specifically in social settings. In this iterated version of the Trust Game, each partner is encountered multiple times. Thus, participants are motivated to learn about how likely it is that an investment with another player yields a monetary gain, while at the same time updating beliefs about their original assessment of the trustworthiness of each of the partners.

We believe some notable strengths of the current study are that we utilized a double-blind, placebo-controlled, within-subject design, and that we replicated many of previous Trust Game findings which have been conducted without pharmacological intervention.

Despite participants playing the game itself twice, and therefore, potentially possessing advance knowledge of the task, particularly when playing for the second time, we nonetheless found robust effects of MPH on trust behavior.

Overall, we showed that MPH administration impacts decision-making in a well-understood economic task, specifically for social game partners who both trigger a low trust belief and who also seldom reciprocate trust over time. We interpret these findings as evidence that MPH can enhance the salience of socially relevant prior beliefs, possibly via a confirmation bias mechanism.

## References

[CR1] Arnsten AFT (2011). Catecholamine influences on dorsolateral prefrontal cortical networks. Biol Psychiatry.

[CR2] Axelrod R, Hamilton WD (1981). The evolution of cooperation. Science.

[CR3] Baayen RH, Davidson DJ, Bates DM (2008). Mixed-effects modeling with cross random effects for subject and items. J Mem Lang.

[CR4] Bates D, Mächler M, Bolker B, Walker S (2015) Fitting linear mixed-effects models using lme4. J Stat Softw 67. 10.18637/jss.v067.i01

[CR5] Berg J, Dickhaut J, McCabe K (1995). Trust, reciprocity and social history. Games Econ Behav.

[CR6] Bódi N, Kéri S, Nagy H, Moustafa A, Myers CE, Daw N, Dibó G, Takáts A, Bereczki D, Gluck MA (2009). Reward-learning and the novelty-seeking personality: a between- and within-subjects study of the effects of dopamine agonists on young Parkinson’s patients. Brain.

[CR7] Camerer CF (2003) Behavioral game theory: experiments in strategic interaction. New York, NY, US: Russell Sage Foundation.

[CR8] Camerer CF, Fehr E (2006) When does “economic man” dominate social behavior? Science (80). 10.1126/science.1110600 10.1126/science.1110600 16400140

[CR9] Campbell-Meiklejohn DK, Simonsen A, Jensen M, Wohlert V, Gjerløff T, Scheel-Kruger J, Møller A, Frith CD, Roepstorff A (2012). Modulation of social influence by methylphenidate. Neuropsychopharmacology.

[CR10] Campbell-Meiklejohn D, Simonsen A, Scheel-Kruger J, Wohlert V, Gjerloff T, Frith CD, Rogers RD, Roepstorff A, Moller A (2012). In for a penny, in for a pound: methylphenidate reduces the inhibitory effect of high stakes on persistent risky choice. J Neurosci.

[CR11] Chang LJ, Sanfey AG (2013). Great expectations: neural computations underlying the use of social norms in decision-making. Soc Cogn Affect Neurosci.

[CR12] Chang LJ, Doll BB, van ‘t Wout M, Frank MJ, Sanfey AG (2010). Seeing is believing: trustworthiness as a dynamic belief. Cogn Psychol.

[CR13] Clatworthy PL, Lewis SJG, Brichard L, Hong YT, Izquierdo D, Clark L, Cools R, Aigbirhio FI, Baron J-C, Fryer TD, Robbins TW (2009). Dopamine release in dissociable striatal subregions predicts the different effects of oral methylphenidate on reversal learning and spatial working memory. J Neurosci.

[CR14] Collins AGE, Frank MJ (2013). Cognitive control over learning: creating, clustering, and generalizing task-set structure. Psychol Rev.

[CR15] Delgado MR, Frank RH, Phelps EA (2005). Perceptions of moral character modulate the neural systems of reward during the trust game. Nat Neurosci.

[CR16] Delgado MR, Miller MM, Inati S, Phelps EA (2005). An fMRI study of reward-related probability learning. Neuroimage.

[CR17] DeVito EE, Blackwell AD, Kent L, Ersche KD, Clark L, Salmond CH, Dezsery AM, Sahakian BJ (2008). The effects of methylphenidate on decision making in attention-deficit/hyperactivity disorder. Biol Psychiatry.

[CR18] Dolder PC, Müller F, Schmid Y, Borgwardt SJ, Liechti ME (2018). Direct comparison of the acute subjective, emotional, autonomic, and endocrine effects of MDMA, methylphenidate, and modafinil in healthy subjects. Psychopharmacology.

[CR19] Doll BB, Jacobs WJ, Sanfey AG, Frank MJ (2009). Instructional control of reinforcement learning: a behavioral and neurocomputational investigation. Brain Res.

[CR20] Doll BB, Hutchison KE, Frank MJ (2011). Dopaminergic genes predict individual differences in susceptibility to confirmation bias. J Neurosci.

[CR21] Eisenegger C, Naef M, Linssen A, Clark L, Gandamaneni PK, Müller U, Robbins TW (2014). Role of dopamine D2 receptors in human reinforcement learning. Neuropsychopharmacology.

[CR22] Fallon SJ, van der Schaaf ME, ter Huurne N, Cools R (2017). The neurocognitive cost of enhancing cognition with methylphenidate: improved distractor resistance but impaired updating. J Cogn Neurosci.

[CR23] Fehr E, Schmidt K (1999). A theory of fairness, competition, and cooperation. Q J Econ.

[CR24] Frank MJ, Seeberger LC, O’reilly RC (2004). By carrot or by stick: cognitive reinforcement learning in parkinsonism. Science.

[CR25] Frank MJ, Scheres A, Sherman SJ (2011) Understanding decision-making deficits in neurological conditions: insights from models of natural action selection. Model Nat Action Sel:330–362. 10.1017/CBO9780511731525.019 10.1098/rstb.2007.2058 PMC2440777 17428775

[CR26] Hysek CM, Simmler LD, Schillinger N, Meyer N, Schmid Y, Donzelli M, Grouzmann E, Liechti ME (2014). Pharmacokinetic and pharmacodynamic effects of methylphenidate and MDMA administered alone or in combination. Int J Neuropsychopharmacol.

[CR27] King-Casas B, Tomlin D, Anen C, Camerer CF, Quartz SR, Montague PR (2005). Getting to know you: reputation and trust in a two-person economic exchange. Science.

[CR28] Krueger F, McCabe K, Moll J, Kriegeskorte N, Zahn R, Strenziok M, Heinecke A, Grafman J (2007). Neural correlates of trust. Proc Natl Acad Sci.

[CR29] Maoz H, Tsviban L, Gvirts HZ, Shamay-Tsoory SG, Levkovitz Y, Watemberg N, Bloch Y (2014). Stimulants improve theory of mind in children with attention deficit/hyperactivity disorder. J Psychopharmacol.

[CR30] Nickerson RS (1998). Confirmation bias: a ubiquitous phenomenon in many guises. Rev Gen Psychol.

[CR31] Oosterhof NN, Todorov A (2008). The functional basis of face evaluation. Proc Natl Acad Sci.

[CR32] Pedroni A, Eisenegger C, Hartmann MN, Fischbacher U, Knoch D (2014). Dopaminergic stimulation increases selfish behavior in the absence of punishment threat. Psychopharmacology.

[CR33] Pessiglione M, Seymour B, Flandin G, Dolan RJ, Frith CD (2006). Dopamine-dependent prediction errors underpin reward-seeking behaviour in humans. Nature.

[CR34] Rahman S, Robbins TW, Hodges JR, Mehta MA, Nestor PJ, Clark L, Sahakian BJ (2006). Methylphenidate (“Ritalin”) can ameliorate abnormal risk-taking behavior in the frontal variant of frontotemporal dementia. Neuropsychopharmacology.

[CR35] R Core Team (2018) R: A language and environment for statistical computing. R Foundation for Statistical Computing, Vienna, Austria. URL https://www.R-project.org/.

[CR36] Sheehan DV, Lecrubier Y, Sheehan KH, Amorim P, Janavs J, Weiller E, Hergueta T, Baker R, Dunbar GC (1998) The mini-international neuropsychiatric interview (M.I.N.I.): the development and validation of a structured diagnostic psychiatric interview for DSM-IV and ICD-10. J Clin Psychiatry 59 Suppl 20:22-33;quiz 34-57. 10.1016/S0924-9338(99)80239-9 9881538

[CR37] Shiels K, Hawk LW, Reynolds B, Mazzullo RJ, Rhodes JD, Pelham WE, Waxmonsky JG, Gangloff BP (2009). Effects of methylphenidate on discounting of delayed rewards in attention deficit/hyperactivity disorder. Exp Clin Psychopharmacol.

[CR38] Singer T, Kiebel SJ, Winston JS, Dolan RJ, Frith CD (2004). Brain responses to the acquired moral status of faces. Neuron.

[CR39] Smith ME, Farah MJ (2011). Are prescription stimulants “smart pills”? The epidemiology and cognitive neuroscience of prescription stimulant use by normal healthy individuals. Psychol Bull.

[CR40] Swanson JM, Volkow ND (2003) Serum and brain concentrations of methylphenidate: Implications for use and abuse. In Neuroscience and Biobehavioral Reviews. 10.1016/j.neubiorev.2003.08.013 10.1016/j.neubiorev.2003.08.013 14624806

[CR41] Swanson JM, Wigal TL, Volkow ND (2011). Contrast of medical and nonmedical use of stimulant drugs, basis for the distinction, and risk of addiction: comment on Smith and Farah (2011). Psychol Bull.

[CR42] ter Huurne N, Fallon SJ, van Schouwenburg M, van der Schaaf M, Buitelaar J, Jensen O, Cools R (2015). Methylphenidate alters selective attention by amplifying salience. Psychopharmacology.

[CR43] Tye KM, Tye LD, Cone JJ, Hekkelman EF, Janak PH, Bonci A (2010). Methylphenidate facilitates learning-induced amygdala plasticity. Nat Neurosci.

[CR44] Van ‘t Wout M, Sanfey AG (2008) Friend or foe: the effect of implicit trustworthiness judgments in social decision-making. Cognition 108:796–803. 10.1016/j.cognition.2008.07.002 10.1016/j.cognition.2008.07.002 18721917

[CR45] van der Schaaf ME, Fallon SJ, ter Huurne N, Buitelaar J, Cools R (2013). Working memory capacity predicts effects of methylphenidate on reversal learning. Neuropsychopharmacology.

[CR46] Volkow ND, Wang G-J, Fowler JS, Logan J, Franceschi D, Maynard L, Ding Y-S, Gatley SJ, Gifford A, Zhu W, Swanson JM (2002). Relationship between blockade of dopamine transporters by oral methylphenidate and the increases in extracellular dopamine: therapeutic implications. Synapse.

[CR47] Volkow ND, Wang G-J, Fowler JS, Ding Y-S (2005). Imaging the effects of methylphenidate on brain dopamine: new model on its therapeutic actions for attention-deficit/hyperactivity disorder. Biol Psychiatry.

[CR48] Wardle MC, De Wit H (2012). Effects of amphetamine on reactivity to emotional stimuli. Psychopharmacology.

[CR49] Williams LM, Hermens DF, Palmer D, Kohn M, Clarke S, Keage H, Clark CR, Gordon E (2008). Misinterpreting emotional expressions in attention-deficit/hyperactivity disorder: evidence for a neural marker and stimulant effects. Biol Psychiatry.

[CR50] Willis J, Todorov A (2006). First impressions. Psychol Sci.

[CR51] Winston JS, Strange BA, O’Doherty J, Dolan RJ (2002). Automatic and intentional brain responses during evaluation of trustworthiness of faces. Nat Neurosci.

